# Selections and Abstracts

**Published:** 1906-05

**Authors:** 


### *SELECTIONS AND ABSTRACTS.*

### The Conduct of a Normal Labor.

In an article on the subject of the conduct of a normal labor at the Johns Hopkins Hospital, J. Morris Slemons gives much of practical interest in a brief and concise manner, closing with the following

### DIRECTIONS FOR PATIENTS DURING PREGNANCY.

(a) Take as much outdoor exercise as possible, but guard against overtiring yourself.

(b) See that the bowels are moved daily.

(c) On the first day of each month send me an 8-oz. bottle of mixed (night and morning) urine; and for the two months preceding the expected date of confinement, send it on the first and fifteenth days of the month. Be sure to send your name with the specimen.

(d) From the sixth month onward, bathe the nipples night and morning with a solution prepared as follows: Fill a tumbler with equal parts of alcohol and water and add to it a tablespoonful of borax. Keep the solution in a bottle, and apply it by menas of absorbent cotton.

(e) Six weeks before the expected date of confinement, buy a confinement outfit. In this is included everything which will be needed by the nurse and myself, except baby's clothes. At the same time provide two pieces of rubber sheeting, ¾x1 yard and 1x2 yards respectively; a bed-pan; two small round agate basins; a 2-quart fountain-syringe; 25 yards of gauze; and two pieces of cotton batting for making bed-pads, or 4 ready-made sanitary bed-pads.

(f) Send for nurse as soon as labor pains commence, and let her use her judgment in sending for me, unless some emergency arises.

(g) Notify me at once if any of the following symptoms be observed at any time during pregnancy:

1. Scanty urine.
2. Persistent headache.
3. Disturbance of vision.
4. Swelling of feet or face.
5. Loss of blood.
6. Persistent constipation.
7. And also when you feel that anything is not as it should be.

(h) I shall call to see you five or six weeks before you expect to be sick, in order to ascertain your condition and to give you any desired advice.

### DIRECTIONS FOR OBSTETRICAL NURSE.

### *Preparations before Labor.*

(a) See that the patient has procured a confinement outfit, and the other articles called for in Directions for Patients, which include everything you or I shall need, except baby-clothes.

(b) Prepare a sufficient number of sterile bed and vulval pads.

(c) A week before the expected date of confinement, prepare five packages for me, two containing six towels or diapers each; one containing leggings, one containing cotton pledgets, and another gauze sponges. Carefully sterilize and label them.

### *At Time of Labor.*

(a) If pains begin between 7 a.m. and 11 p.m., notify me as soon as possible, so that I may know that labor has commenced and make my plans accordingly. But if labor begins between 11 p.m. and 7 a.m., do not notify me until the pains are strong and frequent, or unless you think it necessary for me to see the patient at once.

(b) At the commencement of labor, prepare two large pitchers full of boiled water, covering them with a clean towel.

(c) When labor has definitely set in, give the patient a warm bath and a soapsuds enema.

(d) Make up the bed on the *left* side.

(e) Procure a piece of oilcloth or an old rug to protect the carpet.

(f) Don't give vaginal douches of any kind.

(g) Don't examine patient vaginally under any circumstances.

(h) Prepare the patient for vaginal examination by placing her upon a Kelly rubber pad, and then wash the genitalia thoroughly with soap and hot water, using cotton pledgets instead of a wash-cloth. Wash from above downwards (towards the anus). Cut the pubic hairs if necessary, then bathe the vulva with a 1-1000 bichloride solution, afterwards covering it with a folded towel soaked in the same solution.

(i) Before a vaginal examination, or when the birth of the child appears imminent, roll the night-gown up over the patient's hips and pin it in position, then put on the obstetrical leggings.

*After Labor.*

(a) As soon as labor is over, cleanse the genitalia with cotton pledgets and water, and then bathe with bichloride solution, after which apply a sterilized vulva pad and place the patient upon a sterilized bed-pad.

(b) Don't use an abdominal binder until after the tenth day, unless otherwise directed.

(c) Change vulval pads as often as necessary, washing the genitalia each time with a 1-4000 bichloride solution.

(d) Take temperature and pulse four times a day (8, 12, 4 and 8), unless otherwise directed, and record upon chart.

(e) Don't catheterize until the bladder is distended, and not until after the patient has failed to urinate in a sitting position.

(f) Give half-ounce of Rochelle salts the morning after labor, and repeat in four hours if not effectual.

(g) Bathe nipples with saturated boracic solution before and after each nursing.

(h) Watch carefully for cracked nipples, and report them to me at once.

(i) *Diet.* First 24 hours, milk, soup, coffee or cocoa, and buttered or soft toast. Second and third days, as above, with the addition of boiled or poached eggs, raw or stewed oysters, and wine-jelly. Fourth and fifth days, as above, with the addition of chicken, sweetbreads, potatoes and rice. And then gradually return to ordinary plain diet.

*Care of Child.*

(a) Leave the baby alone until the mother is cared for, wrapping it in a woolen cloth and putting it in a safe place. (Not upon the mother's bed or upon chairs.)

(b) Wash the eyes with a boracic-acid solution.

(c) Rub the child thoroughly with vaseline or sweet-oil, and then give it a full bath, using castile soap and warm water.

(d) Dress the cord with boracic-acid powder and sterile cotton. Don't change dressing again until necessary.

(e) Wash the child daily in your lap, but don't repeat the full bath until the cord comes off.

(f) *Feeding.* Until the milk appears, nurse three times a day, and don't give any other food, unless directed. After the milk appears, feed the child, except after its bath, *every two hours by the clock,* from 6 or 7 a.m. to 10 or 11 p.m. Time one feeding so that it will come directly after the bath, after which the child may be allowed to sleep for three or four hours if it will.

Do not feed but *once* between bedtime and 6 or 7 a.m.

As soon as the milk appears, write out a schedule for feeding the child, and adhere to it, awakening the child at each feeding-time, if necessary.

Before each nursing, wash out the child's mouth with boracic-acid solution.

After the first three weeks, give one or two bottles of milk a day, no matter how much milk the mother may have.

(g) Weigh the child twice a week, and keep a record of it.

CONTENTS OF CONFINEMENT OUTFIT.

Potassium permanganate.
Oxalic acid.
Boric acid.
Bichloride tablets.
Chloroform.

Green soap.
Vaseline.
Ergotole.
Alcohol.

Any of the following articles not already in the house should be secured:

1 piece of rubber sheeting, ¾x1 yard.
1 piece rubber sheeting, 1x2 yards.
1 bed-pan.
2 small round agate basins.
1 two-quart fountain-syringe.
25 yards absorbent gauze.
2 packages cotton batting.
3 absorbent bed-pads.
Absorbent cotton.
Obstetrical leggings.
Safety-pins, large and small.
Nail-brush.

—*Surgery, Gynecology and Obstetrics,* Feb., 1906.

Dr. Taussig, in an article on Post-Operative Cystitis in Women: its Causes and Prevention, gives the following conclusions:

The chief points in the prophylaxis would therefore be—

1. Try to avoid urine retention by the use of one or several of the following methods: Filling the bladder with sterile water at the conclusion of the operation, injecting boro-glycerin solution into the full bladder, having the patients sit up out of bed as early as the nature of the operation will allow.

2. In the operation, handle the bladder carefully and cover its denuded surface as well as possible before the close.

3. Prevent the introduction of germs from the urethra as far as possible, by using a double catheter such as devised by Rosenstein.

4. Internally, you may give urotropin, helmitol, etc.

5. Above all, wherever catherization has to be continued for some time, irrigate the bladder each time with one or two pints of boric acid solution and continue such irrigations with each catheterization, not merely until the first spontaneous urination, but until there is no longer any residual urine.

---

### BACTERIAL THERAPEUTICS OF ENTERITIS; A NOVEL TREATMENT OF INTESTINAL DISORDERS.

Henry Tissier (*La Tribune médicale,* February 24, 1906) advocates treatment of bowel disorders by the novel method of transformation of the bacterial flora of the intestine from pathogenic forms to the normal flora. The treatment has its foundation in the fact that in culture media containing more than one per cent. of sugar, the addition of the ferment of the albuminoids and the acid ferment of hydrocarbons in combination is capable of arresting the action and the development of another simple ferment (especially of the albuminoids), and that under the same conditions a strong mixed ferment arrests the action and the development of a feeble mixed ferment. These preventive actions are attributable solely to the quantity of acids which are produced in the course of the attack upon the hydrocarbons. Therefore, we may arrest any putrid process by adding to the medium a little sugar and the mixed bacterial ferments, when the latter are not already present. In producing these acids the latter bacterial forms will check simple fermentation, and their action will cease spontaneously when they have produced the acid. There will be no chemical modification or microbian development, as long as nothing intervenes to destroy or neutralize this protective acidity. Applying this to the intestine, the author sought to replace an injurious bacterial vegetation by one indifferent to an organism, such as that of an infant at the breast in a normal condition. In order to arrive at this result, it is necessary to modify the chemical constitution of the intestinal media so as to render it favorable to the development of the preventive species. The waste material of digestion should contain only hydrocarbons, and only the smallest quantity of proteid substances. This can be obtained only from a regimen consisting of sugar, starches, and fats principally; and in which the albuminoids are either suppressed or considerably reduced. Finally, in order that the flora may be composed only of mixed ferments, the most simple and the most rapid method is to introduce pure cultures of these species. It is necessary, therefore, to make a choice, among these, of bacteria which are non-pathogenic. Based upon two years' experience, the following method of treatment is outlined: A strict vegetarian diet is prescribed, especially excluding milk, eggs, and all kinds of meat and fish. In order to hasten the appearance of a preventive intestinal flora, one or two wine glasses are given of a pure culture of bacillus acidi paralactici, or preferably of a symbiosis of this with the bacillus bifidus. These cultures are made in peptonized water (salt, 5 parts; peptone, 10 parts; lactose, 20 parts; in 1,000 parts of water), and are very easily prepared by any one familiar with bacteriological studies. They should, preferably, be given fresh. As soon as these microbes begin to grow, the medium becomes imputrescible. No species, putrefying or pathogenic, is able to become acclimated in it. Certain moulds may develop, however, and give it a bad taste and slowly destroy the protective acid, but this would only occur at the end of several months. During the treatment it is necessary to stop all antiseptics, laxatives, irrigations, suppositories, etc. In the first few days of treatment of enteritis, there may be no evacuation of the bowels, and the abdomen may be tympanitic, and there may be some colics or gastric disorder due to change of regimen, but at the end of four or five days, on the average, the constipation ceases, the abdominal pain and swelling diminish, and the tongue cleans off. The alvine discharges lose their putrid odor, they are no longer attended by false membranes, and become soft, yellow, and are slightly acid. Bacteriological examinations show gradual reappearance of the microbes constituting the normal flora. In cases with persistent diarrhea (not tuberculous), the improvement is less rapid, but it is rare that marked amelioration is not observed within a week. In order that the results may be permanent, it is advised that the treatment shall be continued for several weeks after apparent recovery.

Abstract from editorial of *Collier's Weekly* for November 18, 1905.

### ANALYSIS OF LIQUOZONE.

| | |
|---|---|
| Sulphuric Acid | About nine-tenths of one per cent |
| Sulphurous Acid | About three-tenths of one per cent |
| Water | Nearly ninety-nine per cent |

Sulphuric acid is oil of vitriol. Sulphurous acid is also a corrosive poison. Liquozone is a combination of these two, heavily diluted.

### WILL THE COMPOUND "LIQUOZONE" DESTROY GERMS IN THE HUMAN BODY?

This is, after all, the one overwhelmingly important point for determination; for if it will, all the petty fakery and forgery, the liquid oxygen and Professor Pauli and the mythical medical journals may be forgiven. For more than four months now *Collier's* has been patiently awaiting some proof of the internal germicidal qualities of Liquozone. None has been forthcoming except specious generalities from scientific employees of the company—and testimonials. The value of testimonials as evidence is considered in a later article. Liquozone's are not more convincing than others. Of the chemists and bacteriologists employed by the Liquozone Company there is not one who will risk his professional reputation upon the simple and essential statement that Liquozone taken internally kills germs in the human system. One experiment has been made by Mr. Schoen of Chicago, which I am asked to regard as indicating in some degree a deterrent action of Liquozone on the disease of anthrax. Of two guinea-pigs inoculated with anthrax, one which was dosed with Liquozone survived the other, not thus treated, by several hours. Bacteriologists employed by us to make a similar test failed, because of the surprising fact that the dose as prescribed by Mr. Schoen promptly killed the first guinea-pig to which it was administered. A series of guinea-pig tests was then arranged (the guinea-pig is the animal which responds to germ infection most nearly as the human organism responds), at which Dr. Gradwohl, representing the Liquozone Company, was present, and in which he took part. The report follows:

LEDERLE LABORATORIES,
Sanitary, Chemical and Bacteriological Investigations,
518 FIFTH AVENUE,
NEW YORK CITY,

October 21, 1905.

*Anthrax Test.* Twenty-four guinea-pigs were inoculated with anthrax bacilli, under the same conditions, the same amount being given to each. The representative of the Liquozone people selected the twelve pigs for treatment. These animals were given Liquozone in 5 c. c. doses for three hours. In twenty-four hours all pigs were dead—the treated and the untreated ones.

*Second Anthrax Test.* Eight guinea-pigs were inoculated under the same conditions with a culture of anthrax sent by the Liquozone people. Four of these animals were treated for three hours with Liquozone as in the last experiment. These died also in from thirty-six to forty-eight hours, as did the remaining four.

*Diphtheria Test.* Six guinea-pigs were inoculated with diphtheria bacilli and treated with Liquozone. They all died in from forty-eight to seventy-two hours. Two out of three controls (*i. e.,* untreated guinea pigs) remained alive after receiving the same amount of culture.

*Tuberculosis Test.* Eight guinea-pigs were inoculated with tubercle bacilli. Four of these animals were treated for eight hours with 5 c. c. of a 20 per cent. solution of Liquozone. Four received no Liquozone. At the end of twenty-four days all the animals were killed.

Fairly developed tuberculosis was present in all.

To summarize, we would say that the Liquozone had absolutely no curative effect, but did, when given in pure form, lower the resistance of the animals, so that they died, a little earlier than those not treated.

LEDERLE LABORATORIES.
By Ernst J. Lederle.

Dr. Gradwohl, representing the Liquozone Company, stated that he was satisfied of the fairness of the tests. He further declared that in his opinion the tests had proved satisfactorily the total ineffectiveness of Liquozone as an internal germicide.

But these experiments show more than that. They show that in so far as Liquozone has any effect, it tends to lower the resistance of the body to an invading disease. That is, in the very germ diseases for which it is advocated, *Liquozone may decrease the chances of the patient's recovery with every dose that is swallowed, but certainly would not increase them.*

---

### THE DESMOID REACTION OF SAHLI.

Felix Eichler (*Berliner Klinische Wochenschrift*, No. 48, 1905) draws important conclusions from a series of thirty cases from Ewald's clinic as to the practical value of the new Desmoid Reaction of Sahli in the diagnosis of abnormal conditions of the stomach secretions.

This reaction, as first announced by Sahli during the past year, is founded upon the now thoroughly proved fact that the desmoid or connective tissue of our food is digested solely in the stomach, and only then in the presence of a sufficient quantity each of hydrochloric acid and pepsin.

Sahli uses this fact as a working basis of his method in the following clever manner: A small quantity of powdered methylene blue (about 2 gr.) is encased in a thin rubber membrane, which is puckered and tied tightly at one end with ordinary raw catgut, No. 00, care being taken that all possible egress of the methylene blue is prevented.

This bolus, very much of the shape and consistency of an ordinary pill, is given to the patient immediately following a regular mid-day meal, the constituents of which are entirely indifferent for the test.

If, now, the gastric juice contains approximately normal amounts of HCl and pepsin, the catgut, a desmoid tissue, is digested in the stomach, which results in a freeing of the methylene blue from its small rubber envelope. The powder is absorbed and appears in the urine, with its characteristic color reaction, within a certain definite period. If, on the other hand, the HCl or pepsin be absent or even diminished, the rubber bolus passes through the canal entirely intact, so that the urine remains free from the expected coloration. If the kidneys, from any cause, be suspected of a functional insufficiency, which might possibly interfere with the time limit of the test, potassium iodide can be substituted for the methylene and tested for in the saliva, the principle remaining the same.

The method is suggested and highly recommended by the author as a substitute for the test breakfast, not only because of its simplicity, but also in cases where for any reason the stomach tube is contraindicated or refused by the patient.

Eichler, after a careful and thorough testing of the method *in toto,* draws the following conclusions as to its value:

1. The plan is a simple and harmless one, and commends itself especially to the busy practitioner who would gladly find a satisfactory substitute for the rather cumbersome stomach tubes.

2. The test being made immediately following a regular and full meal, is more likely to give a truer insight into the real status of the stomach secretions than does the artificial test breakfast.

3. A positive reaction within 15 to 20 hours is positive proof that the stomach is secreting approximately normal amounts of HCl and pepsin.

4. A negative reaction, or one in which the urine findings are delayed over twenty hours, would make us conclude that the gastric juice is lacking in either one of its digestive constituents—which one, however, the test fails to prove.

5. No definite conclusion as to a subsequent excess of HCl (a hyperchloridia or a hypersecretion) or the degree of diminution of the acid (a hypochloridia) can be drawn from the test.

6. In cases of hypermotility, in which the stomach empties itself too rapidly to allow of complete digestion of the catgut, a negative reaction might be decidedly misleading.

In conclusion, Eichler welcomes the reaction as a valuable addition to our methods of stomach examination, but with limits well defined and drawn. It lacks in exactness and precision and, in this sense, can by no means be considered as a substitute for the stomach tube and test breakfast.

### PREVENTION OF COMPLICATIONS OF MEASLES.

BY DR. G. PINNA (Illinois Policlinic, September, 1905 ; Ref. Brit. Med. Jour.)

The author, starting from the fact that the complications of measles are more fatal than the original disease, proposes to diminish the mortality by preventing the complications. He gives an account of his investigations into two cases. In each, besides a blood count, there was a bacteriological examination of the feces and skin. In the first case the skin yielded the *Staphylococcus pyogenes albus,* the *Micrococcus candicans,* the *Diplococcus lanceolatus,* the *Sarcina alba,* and the *Staphylococcus pyogenes hemorrhagicus.* In the second he isolated from the skin the *Staphylococcus pyogenes albus,* the *Diplococcus lanceolatus,* the *Sarcina alba,* the *Streptococcus pyogenes,* and the *Micrococcus rosettaceus.* In the latter case, with the intention of provoking local suppuration, he pierced the skin with an aseptic platinum needle on the third day after the fever disappeared, and the patient appeared well. Five or six hours later rigors occurred, and an hour afterwards the temperature was 40° C. Inflammation of the right lung declared itself, and, running a mild course, disappeared in three days. During the course of the pneumonia the expectoration contained a few diplococci. As the patient was isolated, confined to her bed, guarded against chills, and in a hospital where there was no other case of pneumonia, the author suggests that her attack may have been caused by the infection conveyed through the skin from the epidermis by the platinum needle. Hence he deduces that we should guard against any breach of continuity of the skin or mucous membrane during convalescence from measles. The skin should be carefully cleansed, the patient's surroundings disinfected, and the simple cases of measles isolated from those with complications, no great aggregation of sick being permitted under any circumstances. Antiseptic treatment of the skin is especially necessary when peeling is taking place, so that there may be no inhalation of micro-organisms from the skin. Particular care should be exercised in avoiding all danger of tuberculosis or diphtheritic infection, and where diphtheria and measles are epidemic at the same time, sufferers from measles should be immunized by the injection of antidiphtheritic serum.

